# The growth rate and clinical outcomes of radiation induced meningioma undergoing treatment or active monitoring

**DOI:** 10.1007/s11060-021-03761-3

**Published:** 2021-04-22

**Authors:** Conor S. Gillespie, Abdurrahman I. Islim, Basel A. Taweel, Christopher P. Millward, Siddhant Kumar, Nitika Rathi, Shaveta Mehta, Brian J. Haylock, Nicola Thorp, Catherine E. Gilkes, David D. A. Lawson, Samantha J. Mills, Emmanuel Chavredakis, Jibril Osman Farah, Andrew R. Brodbelt, Michael D. Jenkinson

**Affiliations:** 1 Institute of Systems, Molecular and Integrative Biology, University of Liverpool, Liverpool, UK; 2 The Walton Centre NHS Foundation Trust, Liverpool, UK; 3 Clatterbridge Cancer Centre NHS Foundation Trust, Liverpool, UK; 4 School of Medicine, University of Liverpool, Cedar House, Ashton Street, Liverpool, L69 3GE UK

**Keywords:** Meningioma, Radiation, Volumetric growth, Natural history, Radiation induced meningioma

## Abstract

**Introduction:**

Radiation induced meningioma (RIM) incidence is increasing in line with improved childhood cancer survival. No optimal management strategy consensus exists. This study aimed to delineate meningioma growth rates from tumor discovery and correlate with clinical outcomes.

**Methods:**

Retrospective study of patients with a RIM, managed at a specialist tertiary neuroscience center (2007–2019). Tumor volume was measured from diagnosis and at subsequent interval scans. Meningioma growth rate was determined using a linear mixed-effects model. Clinical outcomes were correlated with growth rates accounting for imaging and clinical prognostic factors.

**Results:**

Fifty-four patients (110 meningiomas) were included. Median duration of follow-up was 74 months (interquartile range [IQR], 41–102 months). Mean radiation dose was 41 Gy (standard deviation [SD] = 14.9) with a latency period of 34.4 years (SD = 13.7). Median absolute growth rate was 0.62 cm^3^/year and the median relative growth rate was 72%/year. Forty meningiomas (between 27 patients) underwent surgical intervention after a median follow-up duration of 4 months (IQR 2–35). Operated RIMs were clinically aggressive, likely to be WHO grade 2 at first resection (43.6%) and to progress after surgery (41%). Median time to progression was 28 months (IQR 13–60.5). A larger meningioma at discovery was associated with growth (HR 1.2 [95% CI 1.0–1.5], *P* = 0.039) but not progression after surgery (HR 2.2 [95% CI 0.7–6.6], *P* = 0.181). Twenty-seven (50%) patients had multiple meningiomas by the end of the study.

**Conclusion:**

RIMs exhibit high absolute and relative growth rates after discovery. Surgery is recommended for symptomatic or rapidly growing meningiomas only. Recurrence risk after surgery is high.

**Supplementary Information:**

The online version contains supplementary material available at 10.1007/s11060-021-03761-3.

## Introduction

Radiation induced meningioma (RIM) is defined as a meningioma occurring secondary to radiation treatment that satisfies specific criteria relating to: location of radiation, differing histology from previous malignancy, and at least a 5-year interval period after radiation treatment [1–4]. Meningiomas are the most common type of brain tumor to occur following cranio-spinal radiotherapy [5], with a one in eight risk of developing a RIM by the age of 40 [6–8]. Improvements in childhood and adult cancer survival rates have led to an increased incidence of RIM. The management strategy for these patients is an important clinical problem [9].

RIMs are more clinically aggressive than sporadic meningioma, likely to be multiple and recur after surgery [10]. No reported studies have examined the growth rate and outcomes of untreated RIM. MRI screening for late intracranial effects of childhood radiotherapy, including the development of meningioma, is a common clinical practice [11, 12]. Volumetric growth patterns of RIM and the subsequent optimal management of asymptomatic cases remains unclear—a fact highlighted as a physician-reported barrier to screening by 74% of responders in a recent international survey [11].

Volumetric studies of meningioma growth are lacking in general, with many focussing on growth following surgical management [13–17]. A volumetric study conducted from diagnosis will be more relevant for both the patient and clinician, particularly as there are increasing numbers of patients with ‘incidental’ meningiomas undergoing active monitoring [18, 19].

## Objective

To investigate the volumetric growth rates, prognostic factors and outcomes of treated and untreated radiation-induced meningiomas.

## Methods

### Study design, setting and participants

Between 1st January 2007 and 31st March 2019, a single center, retrospective cohort study was performed. The study was approved by the hospital audit committee. Adults > 16 years diagnosed with a meningioma who had received cranial radiation treatment > 5 years before discovery/presentation were eligible for inclusion. Patients with syndromic meningiomas were excluded. The study setting was a tertiary neuroscience center, where a formal screening program for late effects of radiation is not in place. Patients were identified either incidentally or through symptomatic presentation.

### Baseline characteristics

Baseline clinical characteristics included indication for prior radiation therapy, radiation dose and fractionation, latency period (time in years from cranial radiation to first MRI diagnosis of RIM), presenting symptoms (defined as symptomatic if presenting with clinical signs or symptoms attributable to meningioma), age at meningioma diagnosis and sex. Imaging features were single or multiple meningioma, location as per the International Consortium on Meningioma classification [18], volume and signal intensity on T2 MRI (hypo/iso/hyper). Tumor volume was calculated according to the *ABC*/2 formula on contrast-enhanced T1- weighted MRI/CT (*A* = maximum meningioma diameter on axial plane, *B* = diameter perpendicular to *A*, and *C* = maximum height on coronal or sagittal plane). Each tumor was measured 3 times and the mean used to calculate the tumor volume. Dural tails were not included in volume calculation. Inter and intra-rater reliability of meningioma volume was assessed on a random sample of 24 patients (sample size determined using the Bland equation [20]) by 2 observers independently (CSG and BAT) using the intraclass correlation coefficient (ICC).

### Management data

Management decision at diagnosis was stratified into active monitoring or intervention (surgery or radiation therapy). For patients placed under active monitoring, follow-up intervals, tumor volume on each scan and neurological status were recorded, until intervention or end of study period. Surgical outcomes included extent of resection (complete [Simpson grade 1–3] or subtotal [Simpson grade 4–5]), and WHO grade.

### Study endpoints

#### Primary endpoint

The study primary endpoint was volumetric meningioma growth (absolute growth rate (AGR) ≥ 2 cm^3^/year or AGR  ≥ 1 cm^3^/year and relative growth rate (RGR) ≥ 30%). Three MRI scans, and a minimum of 5 months follow-up, were required for a patient to be included in this analysis.

#### Secondary endpoints

Secondary endpoints were treatment (surgery, radiotherapy or stereotactic radiosurgery [SRS]), progression following treatment, development of new meningiomas (multiplicity) and mortality.

### Statistical analysis

#### Volumetric growth rate

Meningioma growth rate was determined using a longitudinal linear mixed-effect regression model for meningioma volume with time as the fixed variable and included both the random intercept and slope. Absolute growth rate (AGR) was defined as the increase in volume per year in cubic centimeters***. ***Relative growth rate (RGR) was defined as percentage increase in volume per year***.***

#### Prognostic analyses

The Chi-square test was used to examine statistical differences in outcomes for categorical variables. The Student t-test, Mann–Whitney U test or Kruskal Wallis test were used to examine continuous variables as appropriate. Correlation between baseline variables was evaluated using the Pearson correlation coefficient. Prognostic factors for the study endpoints were delineated using stepwise multivariate proportional hazard regression analysis, incorporating variables with *P*-values ≤ 0.1 on univariate analysis. Data analysis was conducted using R V4.0.2 and SPSS V25 (IBM, Armonk, NY, USA).

## Results

### Baseline, clinical and radiological features

After excluding two patients with a familial syndrome (Neurofibromatosis type 1 and Gorlin-Chaudhary Moss syndrome) 54 patients with a total of 110 intracranial meningiomas were included. Of these, 18 patients with 37 tumors did not have sufficient radiological follow up, and were excluded from the volumetric component of the study. The baseline, clinical and radiological characteristics are shown in Table 1.

**Table 1:** Patient characteristics for 54 patients with 110 Radiation induced meningioma

Baseline characteristics	Value
Total patients	54
Total meningiomas	110
Single (%)	44 (81.5)
Multiple (%)	10 (18.5)
Male (%)	30 (55.6)
Female (%)	24 (44.4)
Median age at discovery (range)	44.5 (18–82)
Mean latency period (SD)	34.4 (13.7)
Female, n (%)	24 (44.4)
Median age at radiation (IQR)	9.4 (1.7–59.9)
Mean radiation dose (SD)	41.01 (15.0)
Fractionations (SD)	20.9 (10.2)

Symptoms	Frequency (%)
Yes	26 (48.1)
No	28 (51.9)
Headache/raised ICP	13 (50.0)
Cranial nerve deficit	7 (26.9)
Seizures/epilepsy	3 (11.5)
Enlarging mass	1 (3.8)
Other	2 (7.6)

Reason for prior radiation treatment	Frequency (%)
Medulloblastoma	10 (18.5)
Pilocytic astrocytoma	7 (13.0)
Acute lymphoblastic leukaemia (ALL)	7 (13.0)
Other/unknown leukaemia	7 (13.0)
Other/unknown	23 (42.6)

Meningioma laterality	Frequency (%)
Right sided	46 (44.2)
Left sided	47 (45.2)
Central	10 (10.6)

ICOM location	Frequency (%)
Convexity	52 (47.3)
Parafalcine	16 (14.5)
Sphenoid wing	10 (9.1)
Posterior fossa- lateral and posterior	9 (8.2)
Anterior midline	7 (6.4)
Parasagittal	7 (6.3)
Tentorial	5 (4.5)
Intraventricular	1 (0.9)
Intraosseous	1 (0.9)

Signal intensity	Frequency (%)
Isointense	56 (50.9)
Hypointense	7 (6.4)
Hyperintense	27 (24.5)

*ICOM* International Consortium on Meningioma, *ICP* intracranial pressure

The most common indications for primary radiation treatment were medulloblastoma (18.5%, n = 10/54), pilocytic astrocytoma (13%, n = 7/54), and leukemias (26%, n = 14/54) (Table 1). Median age at time of radiation treatment (RT) for primary pathology was 9.4 years (range 1.7–59.9 years). Of 52 patients with age at radiation available, 28 were under 10 years of age (53.8%), 14 were between 10 and 19 (26.9%), and 10 patients were over 19 years of age (19.2%).

Out of 54 patients, 28 were diagnosed incidentally, and 26 were symptomatic. Forty-four patients had a single meningioma at discovery, and 10 patients had multiple meningiomas (24 between them). The mean tumor volume at diagnosis was 4.9 cm^3^ (SD = 11.8) with no statistical difference in mean volume between meningiomas discovered incidentally compared to symptomatically (*P* = 0.351). Inter- and intra-rater reliability were adequate (Online supplementary table S1).

#### Radiotherapy received at first diagnosis

The radiotherapy dose and fractionation regimes were variable (Online supplementary table S2). No radiotherapy data was available for 37% of patients, most frequently because patients had received radiotherapy at another oncology center. The prescribed radiotherapy dose was in the range 18 Gy to 55 Gy at doses per fraction of 1.67 Gy to 2.4 Gy. Lower doses were used in patients treated for acute lymphoblastic leukemia on historical protocols receiving prophylactic cranial irradiation. Medulloblastoma and germinomas were treated in 2 phases with an intermediate dose to the whole craniospinal axis followed by a boost giving a higher dose to the tumor bed. Focal higher doses were employed for low grade glioma and craniopharyngioma. No correlation was observed between radiation dose and latency period (Pearson correlation coefficient = − 0.355, *P* = 0.055).

### Management plans and outcomes

**Fig. 1:**
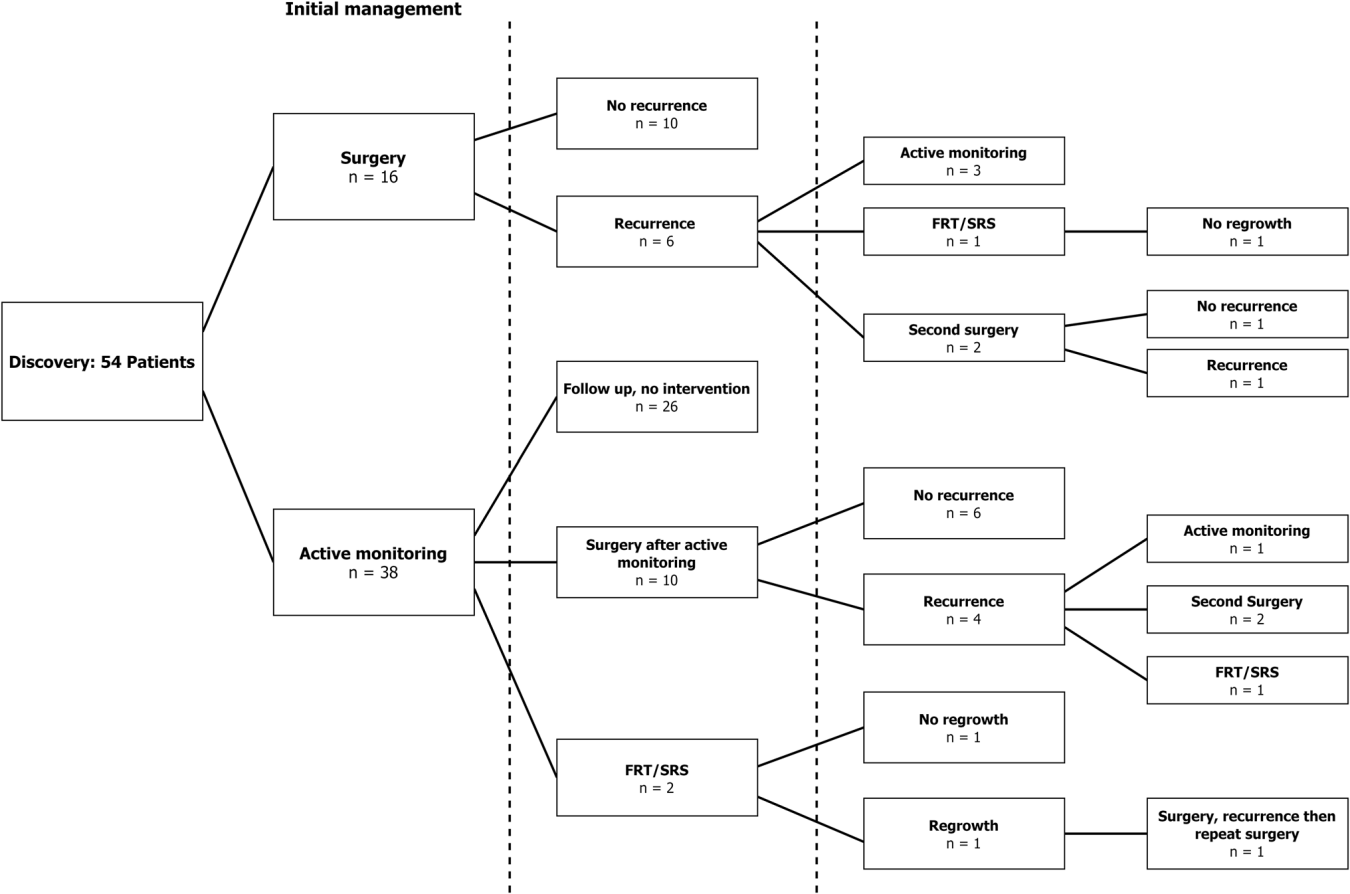
Patient management showing treatment strategy after diagnosis and subsequent management. *RIM* radiation induced meningioma, *FRT* fractionated radiotherapy, *SRS* stereotactic radiosurgery

The management and outcome for patients is shown in Fig. 1. The overall median follow-up period was 74 months after diagnosis (IQR 41–102 months) and twenty-seven patients had surgery for at least one meningioma during the follow up period (n = 27/54, 50%), with 36.4% of all RIMs undergoing surgery (n = 40/110). Among those operated, median time from first scan to operation was 4 months (IQR 2–35).

#### Active monitoring

Thirty-eight patients commenced active monitoring initially (70.4%) (Fig. 1). Median duration of active monitoring was 64.5 months (IQR 24.8–105.8). The median number of scans for each patient was 7 (IQR 4–10). Almost all patients were followed up with 6 monthly or annual contrast enhanced T1- weighted MRI scans. One patient was followed up using CT scans due to having a ferromagnetic surgical clip from previous surgery. Overall, 24 patients developed multiple or additional meningiomas during follow up (44.4%). The median time to discovery of a second or further multiple meningiomas was 53.7 months from time of diagnosis (IQR 25.8–69.3). Of the discovered RIMs, 43 (39.1%) underwent intervention eventually (surgery, n = 40, SRS, n = 2 and *f*RT, n = 1). The indications for intervention were patient preference (n = 3, 7.0%), radiological growth (n = 27, 62.8%) and development of symptoms (n = 13, 30.2%). Symptoms were headaches (n = 6), seizures (n = 2), limb weakness (n = 2), limb sensory change (n = 1), expressive dysphasia (n = 1) and ataxia (n = 1). The median time to surgery from diagnosis was 4 months (IQR 2.0–35.0).

#### Intervention results

Of tumors with Simpson grade available (n = 38), the rate of complete surgical resection was 97.4% (Simpson grade 1, n = 23, Simpson grade 2, n = 13 and Simpson grade 3, n = 1). One patient had a Simpson grade 4 resection. Meningiomas were WHO grade 1 (56.4%), and WHO grade 2 (43.6%) at the time of first operation with no correlation observed with radiation dose (Kruskal Wallis test, *P* = 0.700). Eleven patients progressed (42.3%); 7 were diagnosed on scheduled MRI follow-up and 4 patients had new headache symptoms that prompted an earlier MRI scan. The median time to progression was 28 months (IQR 13–60.5 months). Five patients underwent a second operation for tumor progression. In all re-operated patients, complete surgical resection was achieved and the pathology revealed WHO grade 2 (n = 3), WHO grade 1 (n = 1) and WHO grade 3 (n = 1) meningiomas. Radiation treatment (SRS and *f*RT) was administered after a period of active monitoring (n = 3), and following progression after surgery (n = 2). One patient who underwent SRS continued to demonstrate evidence of meningioma growth, required two operations, and demonstrated malignant transformation from WHO grade 2 to WHO grade 3 at last operation.

### Growth characteristics

**Fig. 2:**
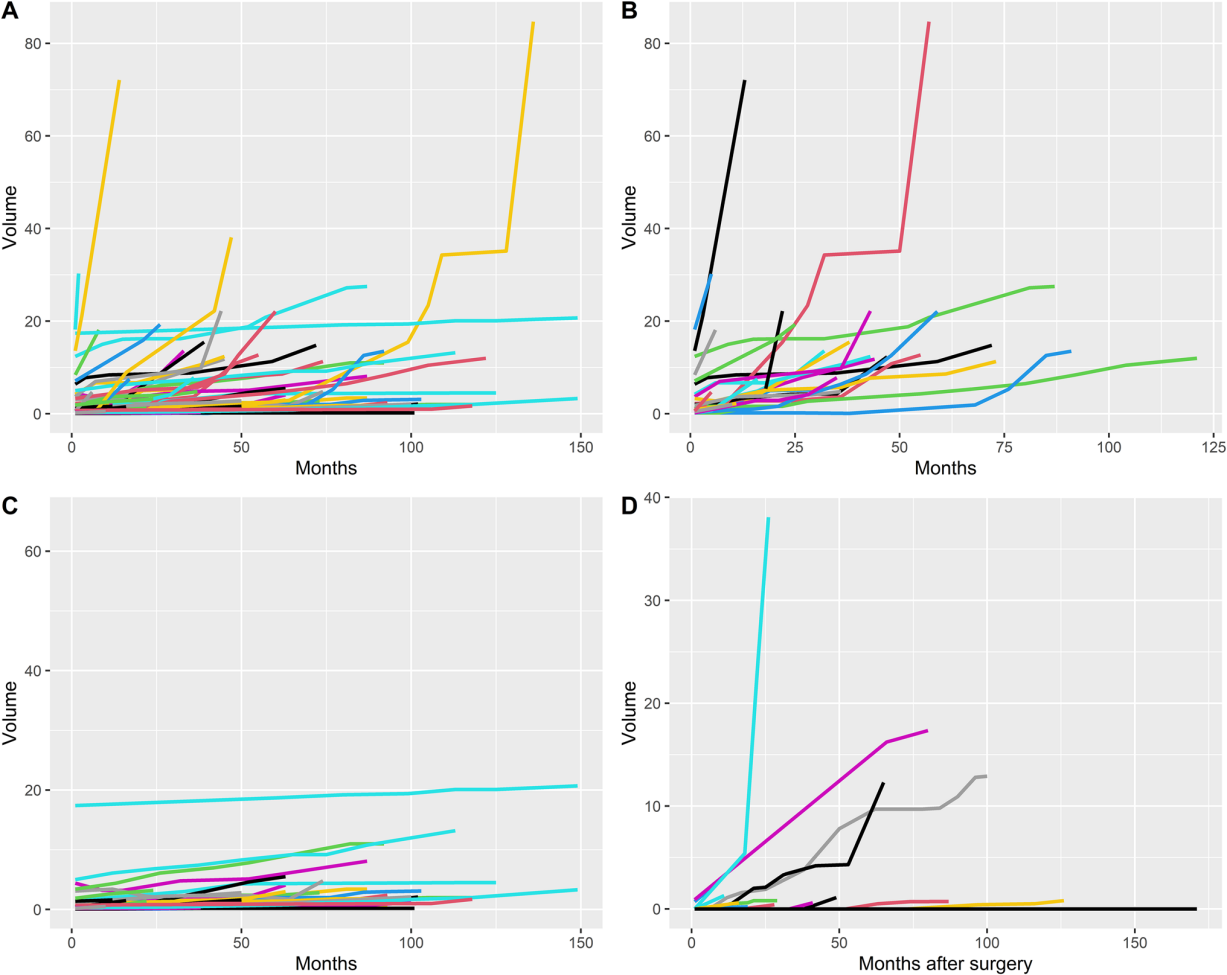
Volume time plots demonstrating. **a** All RIM patients with volumetric analysis. **b** Growth plots of RIMs that met standard growth definition of AGR ≥ 2 cm^3^/year or AGR ≥ 1 cm^3^/year and RGR ≥ 30% per year. **c** Growth plots of RIMs that did not grow during the study period and **d** meningioma growth after surgery

The growth rates are shown in Fig. 2. 39.7% of meningiomas with more than 5 months of clinical follow up demonstrated volumetric growth during the study period (n = 29/73). How this compares to non-RIM tumor growth in other studies is shown in Table 2.

**Table 2:** Table of growth definitions used in meningioma studies, with percentage of RIM meeting each definition

Author and year	Growth definition	Population studied	% of meningiomas that met the study-specific growth definition (n/total)	% of RIM in our study that met growth definition (n/total)
Islim et al. 2020* [19]	AGR ≥ 2 cm^3^/year or AGR ≥ 1 cm^3^/year and RGR ≥ 30%/year	Incidental meningioma	7.5% (29/385)	39.7% (29/73)
Materi et al. 2020 [21]	AGR > 1.28 cm^3^/year	Sub-totally resected meningioma	NA	35.6% (26/73)
Behbahani et al. 2019 [22]	Volume increase > 15% Volume increase > 8.2%	Incidental meningioma	70.6% (72/102) 79.4% (81/102)	95.9% (70/73) 97.3% (71/73)
Lee et al. 2017 [23]	AGR ≥ 2 cm^3^/year	Untreated meningioma (incidental and symptomatic)	25.4% (59/232)	28.7% (21/73)
Lee et al. 2017* [24]	AGR ≥ 2 cm^3^/year or AGR ≥ 1 cm^3^/year and RGR ≥ 30%/year	Untreated meningioma (incidental and symptomatic)	29.7% (69/232)	39.7% (29/73)
Hunter et al. 2017 [25]	Volume increase > 20%	Sub-totally resected petroclival meningioma	66.7% (15/23)	94.5% (69/73)
Hashimoto et al. 2012 [26]	Volume increase > 15%	Incidental meningioma	62.8% (71/113)	95.9% (70/73)
Nakasu et al. 2011 [27]	Volume increase > 8.2%	Incidental and residual/recurrent meningioma	84.6% (44/52)	97.3% (71/73)
Oya et al. 2011 [28]	Volume increase > 8.2%	Untreated meningioma (incidental and symptomatic)	44% (120/273)	97.3% (71/73)
Hashiba et al. 2009 [29]	Volume increase > 15%	Incidental meningioma	62.9% (44/70)	95.9% (70/73)
Other criteria:	20% change per year 33% change per year ≥ 1 cm^3^ per year			80.8% (59/73) 68.5% (50/73) 38.4% (28/73)

*AGR* absolute growth rate, *RGR* relative growth rate

*Studies which used the same meningioma growth definition

The median absolute growth was 2.1 cm^3^ (IQR 0.8–8.1), and the median absolute growth rate was 0.62 cm^3^ per year (IQR 0.24–2.29). The median relative growth rate (RGR) was 246% (IQR 113–586%), and the median relative volume growth per year was 72% (IQR 29–245%). The overall median tumor doubling time (TDT) was 918 days.

Meningiomas that had surgical intervention grew at a significantly faster rate (median TDT 544 days vs 2301 days, *P* < 0.001) and meningiomas that were asymptomatic grew at a slower rate (median TDT 1921 days vs 1104 days), although this was not statistically significant (*P* = 0.506). Of the operated meningiomas, there was a difference between the doubling times of WHO grade 1 and WHO grade 2 tumors (median TDT 460 days vs 280 days respectively), however this was not statistically significant (*P* = 0.395).

### Prognostic factors for study outcomes

**Fig. 3:**
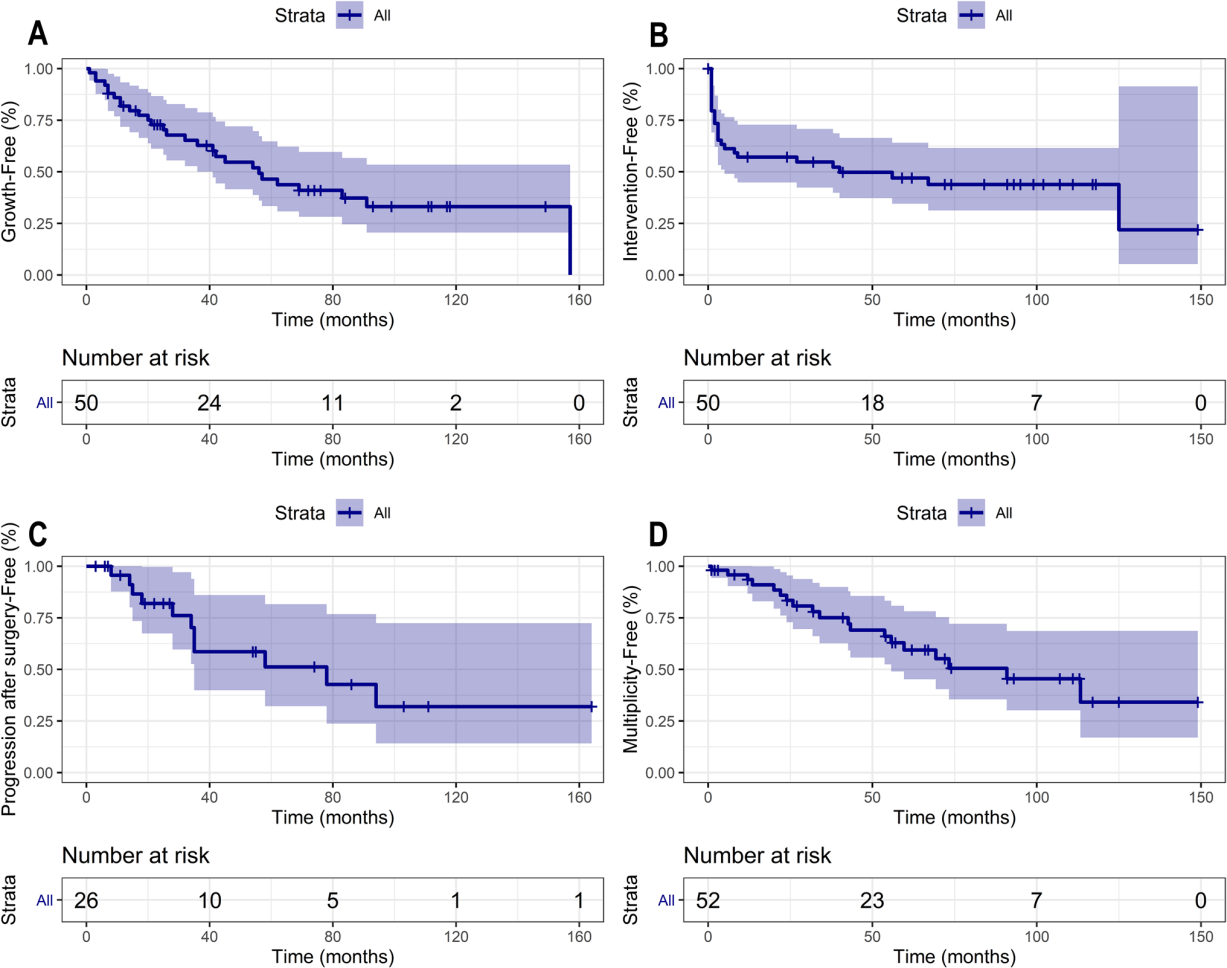
Kaplan Meier curves demonstrating Kaplan Meier curves demonstrating. **a** Growth-Free survival, **b** intervention-free survival, **c** progression-free survival after surgery, and **d** multiple-free survival in RIM patients

Median meningioma growth free survival was 56 months (95% CI 41–91). Median intervention free survival was 40 months (95% CI 5-NA). Median progression free survival after surgery was 78 months (95% CI 38-NA), and the median multiplicity free survival was 69 months after diagnosis (95% CI 42.6-NA) (Fig. 3).

Prognostic factors for tumor growth, surgery and recurrence are shown in online supplementary material tables S3–S7. Tumors that demonstrated ‘growth’ according to the definition during the study period were associated with a higher risk of requiring surgery (HR 14.6 [95% CI 3.4–62.6], *P* < 0.001).

On univariate analysis, factors associated with growth were: tumors causing new neurological symptoms (HR 4.1 [95% CI 1.2–13.7], *P* = 0.022), and larger tumor volume at diagnosis (HR 1.2 [95% CI 1.0–1.5], *P* = 0.030). Increased age at diagnosis was associated with reduced risk of growth (HR 0.95 [95% CI 0.9–1.0], *P* = 0.044). Factors associated with surgery were a symptomatic presentation (HR 8.1, 95% CI [2.2–29.5], *P* = 0.002), T2 hyperintensity (HR 3.6 [95% CI 1.4–9.3], *P* = 0.007), meeting the growth definition (HR 55.1 [95% CI 10.7–282.9], *P* < 0.001), and increased tumor volume (HR 1.1 [95% CI 1.0–1.1], *P* = 0.041). There were no factors identified that were significantly associated with progression after surgery or development of multiple meningioma. On multivariate analysis, large meningioma volume remained a significant factor for meningioma growth (HR 1.2 [95% CI 1.0–1.5], *P* = 0.039).

### Overall patient outcomes

The majority of patients were alive at the end of the study period (96.3%, n = 52/54). One patient died due to a myocardial infarction whilst under follow up, and the other patient had an existing diagnosis of recurrent hemangioblastoma that was being managed palliatively at the time of RIM discovery. Half of the study cohort had multiple meningiomas by the end of the study period or at 5 years after diagnosis (50%, n = 27/54). Two patients developed 8 new RIM during the study period.

## Discussion

In this study, we highlight that on long-term follow up, RIM demonstrate high absolute and relative growth rates. Symptomatic presentation and large volume tumors were associated with higher rates of tumor growth. Radiation induced meningioma exhibited a high rate of early recurrence after surgical resection compared to sporadic meningioma [30].

This cohort of RIM patients appears to have less of a female predominance than sporadic meningioma [31]. This is congruent with previous knowledge [10, 32], and it has been suggested that males have an excess risk of developing RIM, due to increased susceptibility to NF2 rearrangements after radiation treatment [33, 34].

The finding that half patients develop multiple RIMs over the course of their lifetime is in contrast to recent studies that reported lower rates (11.9–15.8%) [1, 10]. The propensity to develop new meningiomas warrants the need for long-term follow-up and more frequent interval imaging (e.g. 6 monthly) may be required [35]. Our recurrence rate (41.0%) after surgery is higher than the published literature (18.3%) [36], as is the proportion of RIMs with a WHO grade 2 histology (43.6%) [1, 37].

Due to the small number of patients undergoing stereotactic radiosurgery (SRS) and fractionated radiation therapy (fRT) in our series, the benefit of SRS and further fRT remains unclear. A recent study investigating the use of SRS in RIM concluded that SRS may be effective in WHO grade 1 or incidentally diagnosed radiation induced meningiomas [38, 39], however it is worth noting that lower volume RIM in our cohort were less likely to grow, and therefore treatment response to SRS may be overstated.

Two previous studies have defined a fast-growing non-radiation induced meningioma as one which demonstrates an AGR ≥ 2 cm^3^/year or AGR ≥ 1 cm^3^/year and RGR ≥ 30%/year. These were a study of 441 incidental meningiomas discovered over a similar time frame in our center, and a study of 232 untreated incidental and symptomatic meningiomas. The results of these two studies, which utilised two different methods of volume measurement; ABC/2 and manual segmentation, both demonstrated a lower risk of sporadic meningioma growth in comparison to RIMs (7.5% and 29.7% vs 39.7%) (Table 2), supplementing the conclusion of RIMs being clinically aggressive meningiomas. These findings also serve to highlight the need for standardized growth definitions in meningioma. Investigators use a variety of definitions to define growth. In order to effectively determine what constitutes meningioma ‘growth’, uniform criteria need to be established to validate volumetric study findings. The Response Assessment in Neuro-Oncology (RANO) working group recently emphasized the need to consider time interval, size and imaging modality as variables in assessing meningioma growth, and proposed a fast-growing meningioma as one that has demonstrated a > 15% increase in bidimensional enhancing product in the previous 6 months [40]. These criteria remain to be validated in both sporadic and radiation induced meningiomas.

It has been suggested that RIM tumorigenesis occurs through different mechanisms than sporadic meningioma [41]. NF2 mutations have been reported to occur less frequently in RIMs (6% vs 30–50%), and other druggable targets found in sporadic meningioma (SMO, TRAF7, KLF4, PIK3CA and ATK1) are often absent in RIMs [33, 42]. Certain copy number alterations (CNAs) have been observed almost universally in RIM, such as combined loss of heterozygosity on chromosome 1p and 22q, which is less commonly encountered in benign meningioma [43, 44]. This supports the hypothesis that radiation triggers genome structural rearrangements through error prone repair of double strand DNA breaks, which may lead to non-homologous end joining and increased growth potential. It is postulated that craniospinal radiation may also lead to NF2 intronic rearrangements/fusion events (found in 39% RIM vs 0% sporadic meningioma) as opposed to mutations seen in sporadic meningioma, subsequently leading to inactivation.

Our clinical findings correlate with these factors and indicate that RIM appears to be a distinct molecular entity with unique growth properties, and the drivers behind this need to be investigated by molecular studies in future work.

Harrison et al. classified RIMs into three groups based on amount of radiation administered, with low dose defined as < 10 Gy, moderate dose 10–20 Gy, and high dose > 20 Gy [45]. The vast majority of our cases represent RIMs that occurred as a result of high dose RT. In comparison to those due to low dose RT (for historical treatments such as tinea capitis), RIMs arising from high dose RT tend to exhibit a shorter latency period, a more even male to female ratio, and increased recurrence after surgery [36, 45–47].

### Study strengths

To our knowledge, this is the first study to analyse the volumetric growth rates of RIM and their natural history, the first to examine volumetric growth rates immediately after tumor discovery, and the potential prognostic factors for growth, surgery, progression, and development of multiple RIM. Furthermore, our cohort of RIMs was diagnosed between 2007 and 2019 and represents a more recent case-series with a median follow up period of 6 years.

### Study limitations

There are several limitations to our study. First, the study was retrospective in design and not all tumor volumes were available to be comprehensively measured during the study period, and the regression analyses were limited to tumors that underwent more than one follow-up MRI scan. Second, volumetric analysis was commenced upon tumor discovery, and therefore it is not possible to delineate for how long the tumor was present before being discovered, in addition to its growth rate before discovery. There was therefore no standardized diagnostic start point for the study. Third, the use of intervention as a study endpoint was limited by clinician and patient biases and might have impacted the results of the study. The tumor board in our center considers the clinical and radiological status of the meningioma, patient comorbidities and performance status before discussion of the recommended and alternate management strategies with the patient to reach a shared-care decision. Due to the retrospective nature of the study, we were unable to assess the reasons behind continued monitoring in cases of symptomatic progression. Nonetheless, it is reasonable that this was due to patient preference, personal and social circumstances, loss of driving license for at least 6 months in the UK and risks of unemployment, post-surgical epilepsy, new neurological deficit and death. Fourth, due to lack of screening for late effects of cranial irradiation in our center, patients with more indolent RIMs may have not been detected clinically, and thus our cohort may consist of more clinically aggressive RIMs than if all childhood cancer survivors were systematically screened. Fifth, assessment of whether the meningioma occurred within the radiation field or not was not feasible. This was due to the inability to access the planning scans for patients who mostly underwent intracranial radiation between 1970 and 1990. Nonetheless, radiation techniques at the time were mostly whole brain radiotherapy and two-dimensional conformal radiotherapy, which is associated with out of field spillage [48]. Therefore, it’s likely that patients in this study are a true representative of RIMs. Additionally, due to the historic nature of radiation details, number of fractions and total radiation dose were missing for more than a third of patients. Finally, in this study, we used an approximated method of measuring tumor volume (ABC/2), however this has been externally validated to be a reliable method of measuring tumor volume in meningiomas [49, 50].

## Conclusions

Radiation induced meningioma is a less commonly reported, but increasing clinical problem with no clear consensus on the optimal management strategy. RIM are more likely to be symptomatic, require surgical resection, have grade 2 histology, and progress compared to sporadic meningioma. They exhibit high absolute and relative growth, and thus appear to be more likely to develop clinical and radiological progression following discovery.

## Supplementary Information

Below is the link to the electronic supplementary material.

Supplementary file1 (DOCX 55 kb)

## Data Availability

Anonymized data are available (upon reasonable request) from the corresponding author.
