# Supplementary material for: The growth rate and clinical outcomes of radiation induced meningioma undergoing treatment or active monitoring
Source: J Neurooncol. 2021 Apr 22;153(2):239–49. doi: 10.1007/s11060-021-03761-3 (PMC8211577; doi:10.1007/s11060-021-03761-3)
Supplement: Supplementary file 1 — Supplementary file1 (DOCX 55 kb) [file 11060_2021_3761_MOESM1_ESM.docx]

**Online only supplementary material**

Title: The growth rate and clinical outcomes of radiation induced meningioma undergoing treatment or active monitoring

Journal of Neuro-Oncology

Authors: Gillespie CS, Islim AI, Taweel BA, Millward CP, Kumar S, Rathi R, Mehta S, Haylock B, Thorpe N, Gilkes CE, Lawson DA, Mills SJ, Chavredakis E, Farah JO, Brodbelt AR & Jenkinson MD.

Corresponding author email address: [hlcgill2@liv.ac.uk](mailto:hlcgill2@liv.ac.uk)


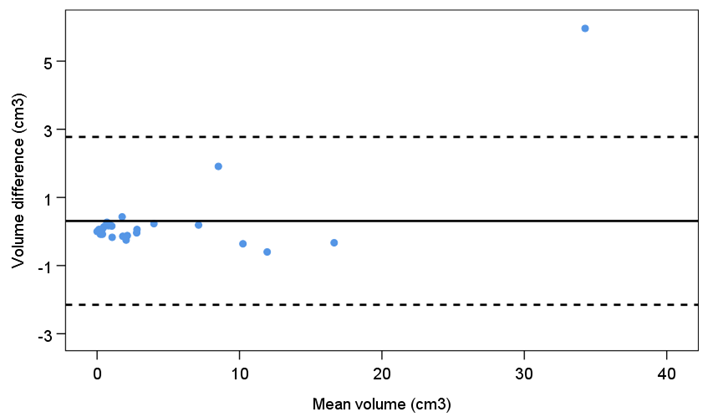


Supplementary Figure S1: Bland-Altman plot showing agreement between independent volumetric measurements by authors CG and BT.

| **Meningioma volume Intraclass correlation coefficient (95% CI)** | |
| --- | --- |
| Inter-rater variability^A^ | Intra-rater variability^B^ |
| 0.993 (95% CI 0.984-0.997) | 0.998 (95% CI 0.997-0.999) |

^A^Set to two-way mixed

^B^Set to one-way random

Supplementary table S1: Intraclass correlation coefficient (ICC) with a 95% Confidence interval displaying inter-rater variability and intra-rater variability between two independent authors, CSG and BAT.

| **Patient number** | **Primary RT reason** | **FRT dose (Gy)** | **Fractionation** | **Age at FRT** | **Age at discovery (years)** | **Latency period (years)** | **RIM localization** | **ICOM location** | **Multiple (yes/no)** |
| --- | --- | --- | --- | --- | --- | --- | --- | --- | --- |
| 1 | Pilocytic Astrocytoma | 42.5 | 20 | 15.0 | 50.1 | 35.1 | Right | Convexity | No |
| 2 | Hemangioblastoma | NA | NA | 1.7 | 48.8 | 47.1 | Right | Parasagittal | No |
| 3 | Ependymoma | NA | NA | 5.5 | 61.0 | 56.5 | Left | Frontal convexity | No |
| 4 | Medulloblastoma | NA | NA | 6.9 | 28.7 | 23.8 | Right | Parafalcine | Yes |
| 5 | Medulloblastoma | 45 | 20 | 8.7 | 51.4 | 41.1 | Left | Anterior midline | Yes |
| 6 | Other glioma | NA | NA | 6.7 | 48.0 | 41.3 | Left | Posterior fossa- lateral and posterior | No |
| 7 | Unknown | NA | NA | 2.0 | 47.0 | 45.0 | Right | Convexity | Yes |
| 8 | Hemangioblastoma | 45 | 20 | 13.3 | 48.9 | 35.6 | Left | Posterior fossa- lateral and posterior | No |
| 9 | NHL | 36 | 20 | 57.7 | 75.6 | 17.9 | Right | Sphenoid wing | No |
| 10 | ALL | 24 | 10 | 18.2 | 45.1 | 27.3 | Right | Convexity | No |
| 11 | Acromegaly/Pituitary Adenoma | NA | NA | 42.0 | 72.1 | 29.1 | Midline | Anterior midline | No |
| 12 | Other glioma | 54 | 30 | 56.9 | 65.7 | 8.8 | Left | Convexity | No |
| 13 | Pilocytic Astrocytoma | 45 | 21 | 14.8 | 42.7 | 27.9 | Left | Convexity | Yes |
| 14 | Other glioma | 43.5 | NA | 10.2 | 37.9 | 27.7 | Left | Convexity | Yes |
| 15 | Acromegaly/Pituitary Adenoma | 45 | 20 | 34.8 | 63.8 | 29.0 | Midline | Parafalcine | No |
| 16 | Medulloblastoma | 60  30* | 40  20* | 17.4 | 40.9 | 28.3 | Right | Convexity | Yes |
| 17 | Other/Unknown Leukemia | NA | NA | 3.0 | 42.0 | 39.0 | Left | Anterior midline | Yes |
| 18 | Germinoma | 40 | 25 | 9.5 | 23.4 | 12.9 | Left | Convexity | Yes |
| 19 | Other/Unknown Leukemia | 24 | 15 | 9.6 | 46.0 | 36.4 | Left | Parafalcine | Yes |
| 20 | Ependymoma | NA | NA | 9.0 | 41.5 | 32.5 | Right | Convexity | Yes |
| 21 | Other/Unknown Leukemia | NA | NA | NA | 41 | NA | Midline | Intraosseous | Yes |
| 22 | Pilocytic Astrocytoma | 42.5 | 20 | 7.9 | 38 | 22.8 | Left | Sphenoid wing | No |
| 23 | Medulloblastoma | 50 | 30 | 6.9 | 29 | 22.4 | Left | Convexity | No |
| 24 | Other/Unknown Leukemia | 50 | 30 | NA | 49 | 44.1 | Right | Parasagittal | No |
| 25 | ALL | 18 | 9 | 3.5 | 29 | 25.5 | Midline | Anterior midline | No |
| 26 | Medulloblastoma | NA | NA | 7.0 | 69 | 62 | Right | Tentorial | Yes |
| 27 | Pilocytic Astrocytoma | 45 | 20 | 13.0 | 34 | 21.4 | Midline | Parafalcine | No |
| 28 | Pilocytic Astrocytoma | 45 | 20 | 4.1 | NA | NA | Right | Convexity | Yes |
| 29 | Medulloblastoma | 55 | 33 | 1.9 | 20 | 18.8 | Left | Sphenoid wing | No |
| 30 | Other | NA | NA | 14.0 | 47 | 14 | Left | Tentorial | No |
| 31 | Other glioma | 45 | 20 | 18.6 | 37 | 19.5 | Right | Convexity | Yes |
| 32 | Medulloblastoma | NA | NA | 1.8 | 16 | 14.0 | Left | Parasagittal | Yes |
| 33 | Acromegaly/Pituitary Adenoma | 45 | 25 | 59.9 | 68.8 | 9.3 | Right | Parafalcine | No |
| 34 | Pilocytic Astrocytoma | 40 | 20 | 10.5 | 39.0 | 28.4 | Midline | Parafalcine | Yes |
| 35 | ALL | 18 | 10 | 4.5 | 28.2 | 23.7 | Midline | Anterior midline | Yes |
| 36 | Medulloblastoma | NA | NA | 2.4 | 26.1 | 23.7 | Left | Posterior fossa- lateral and posterior | No |
| 37 | Craniopharyngioma | 54 | 30 | 26.9 | 50.7 | 23.8 | Right | Intraventricular | Yes |
| 38 | NHL | 18 | 10 | 2.8 | 43.6 | 40.8 | Right | Parasagittal | No |
| 39 | Acromegaly/Pituitary Adenoma | NA | NA | 26.0 | 47.1 | 21.1 | Left | Convexity | Yes |
| 40 | Other glioma | 58 | 30 | 9.2 | 31.2 | 22.0 | Left | Tentorial | Yes |
| 41 | Other/Unknown Leukemia | NA | NA | 10.0 | 52.1 | 42.1 | Left | Convexity | No |
| 42 | Ependymoma | 40 | 20 | 34.9 | 70.3 | 35.4 | Right | Convexity | No |
| 43 | Other | NA | NA | 7.0 | 78.0 | 71.0 | Left | Convexity | Yes |
| 44 | Germinoma | NA | NA | 5.0 | 27.0 | 22.0 | Right | Convexity | No |
| 45 | Germinoma | 45 | 25 | 22.5 | 31.8 | 9.3 | Left | Convexity | No |
| 46 | Pilocytic Astrocytoma | NA | NA | 17.1 | 55.1 | 38.0 | Left | Convexity | Yes |
| 47 | ALL | 24 | 12 | 3.4 | 42.6 | 39.2 | Left | Convexity | No |
| 48 | ALL | 18 | 10 | 2.9 | 36.7 | 33.8 | Midline | Anterior midline | No |
| 49 | ALL | 24 | 12 | 6.2 | 47.2 | 41.0 | Midline | Sphenoid wing | Yes |
| 50 | ALL | 18 | 10 | 11.4 | 42.2 | 30.8 | Left | Parafalcine | Yes |
| 51 | Medulloblastoma | 45 | NA | 10.3 | 34.8 | 24.5 | Left | Convexity | No |
| 52 | Medulloblastoma | 45 | 20 | 4.6 | 39.2 | 34.6 | Midline | Convexity | Yes |
| 53 | Other/Unknown Leukemia | NA | NA | 38.0 | 55.0 | 17.6 | Right | Parafalcine | No |
| 54 | Acromegaly/Pituitary Adenoma | 45 | 25 | 19.3 | 46.0 | 26.7 | Right | Convexity | No |
| *Patient received 60 Gy with surgery and 30 Gy after recurrence 12 months after surgery, **Split into 40 Gy and 20 Gy, NHL= Non-Hodgkin’s Lymphoma, ALL= Acute Lymphoblastic Leukemia, FRT= Fractionated radiotherapy, ICOM= International Consortium on Meningioma, NA= Not available | | | | | | | | | |

Supplementary table S2: Baseline radiation characteristics and indications of 54 RIM patients.

| Univariate analysis | | | |
| --- | --- | --- | --- |
| Volumetric growth | **Risk factor** | **Odds ratio (95% CI)** | **P value*** |
|  | Age at radiation | 0.970 (0.911-1.032) | 0.338 |
|  | Age at discovery | 0.948 (0.900-0.998) | **0.044*** |
|  | Male sex | 1.800 (0.443-7.308) | 0.411 |
|  | Latency period | 0.986 (0.941-1.033) | 0.556 |
|  | Symptomatic presentation | 4.105 (1.230-13.704) | **0.022*** |
|  | Radiation dose | 0.999 (0.946-1.054) | 0.968 |
|  | Fractionations | 1.020 (0.937-1.111) | 0.640 |
|  | Volume at discovery | 1.217 (1.019-1.452) | **0.030*** |
|  | T2 hyperintensity | 2.436 (0.784-7.567) | 0.124 |
|  | Skull base vs non-skull base location | 2.078 (0.742-5.817) | 0.164 |
|  | Multiple meningioma at discovery | 0.938 (0.175-5.022) | 0.940 |

Supplementary table S3: Univariate logistic regression of factors associated with volumetric growth. *Univariate factors incorporated into multivariate analysis if P<0.1 (bold).

| Surgery | **Risk factor** | **Odds ratio (95% CI)** | **P value*** |
| --- | --- | --- | --- |
|  | Age at radiation | 0.988 (0.946-1.032) | 0.580 |
|  | Age at discovery | 0.935 (0.896-0.976) | **0.002*** |
|  | Male sex | 0.750 (0.240-2.341) | 0.620 |
|  | Latency period | 0.947 (0.911-0.985) | **0.007*** |
|  | Symptomatic presentation | 8.075 (2.212-29.477) | **0.002*** |
|  | Radiation dose | 1.024 (0.975-1.075) | 0.345 |
|  | Fractionations | 1.056 (0.963-1.157) | 0.247 |
|  | Volume at discovery | 1.058 (1.002-1.117) | **0.041*** |
|  | T2 hyperintensity | 3.636 (1.417-9.331) | **0.007*** |
|  | Skull base vs non-skull base location | 0.917 (0.367-2.292) | 0.852 |
|  | Multiple meningioma at discovery | 0.655 (0.153-2.804) | 0.568 |
|  | Volumetric growth | 55.125 (10.740-282.941) | **<0.001*** |
|  | Volumetric growth | 0.938 (0.128-6.875) | 0.949 |
|  | Extent of resection | n/a | n/a |
|  | WHO grade | 2.476 (0.610-10.058) | 0.205 |

Supplementary table S4: Univariate logistic regression of factors associated with surgery. *Univariate factors incorporated into multivariate analysis if P <0.1 (bold).

| Progression after surgery | **Risk factor** | **Odds ratio (95% CI)** | **P value*** |
| --- | --- | --- | --- |
|  | Age at radiation | 1.016 (0.950-1.086) | 0.650 |
|  | Age at discovery | 1.031 (0.979-1.086) | 0.254 |
|  | Male sex | 1.085 (0.096-12.315) | 0.948 |
|  | Latency period | 1.058 (0.985-1.138) | 0.124 |
|  | Symptomatic presentation | 0.506 (0.068-3.743) | 0.506 |
|  | Radiation dose | 1.042 (0.874-1.242) | 0.647 |
|  | Fractionations | 1.035 (0.801-1.337) | 0.791 |
|  | Volume at discovery | 0.968 (0.746-1.256) | 0.808 |
|  | T2 hyperintensity | 0.219 (0.020-2.447) | 0.217 |
|  | Skull base vs non-skull base location | 0.282 (0.036-2.234) | 0.231 |
|  | Multiple meningioma at discovery | 6.481 (0.405- 103.824) | 0.187 |
|  | Volumetric growth | 0.938 (0.128-6.875) | 0.949 |
|  | Extent of resection | n/a | n/a |
|  | WHO grade | 2.476 (0.610-10.058) | 0.205 |

Supplementary table S5: Univariate logistic regression of factors associated with progression after surgery. *Univariate factors incorporated into multivariate analysis if P<0.1 (bold).

| Development of multiple meningioma | **Risk factor** | **Odds ratio (95% CI)** | **P value*** |
| --- | --- | --- | --- |
|  | Age at radiation | 0.971 (0.926-1.019) | 0.235 |
|  | Age at discovery | 0.982 (0.941-1.025) | 0.406 |
|  | Male sex | 0.416 (0.131-1.318) | 0.136 |
|  | Latency period | 1.006 (0.961-1.054) | 0.798 |
|  | Symptomatic presentation | 1.429 (0.457-4.465) | 0.540 |
|  | Radiation dose | 1.018 (0.971-1.068) | 0.460 |
|  | Fractionations | 1.013 (0.943-1.089) | 0.719 |
|  | Volume at discovery | 1.007 (0.965-1.051) | 0.750 |
|  | T2 hyperintensity | 1.111 (0.294-4.205) | 0.877 |
|  | Skull base vs non-skull base location | 0.235 (0.046-1.206) | **0.083*** |
|  | Multiple meningioma at discovery | 2.000 (0.495-8.076) | 0.330 |
|  | Volumetric growth | 1.083 (0.255-4.596) | 0.914 |

Supplementary table S6: Univariate logistic regression of factors associated with development of multiple meningioma. *Univariate factors incorporated into multivariate analysis if P<0.1 (bold).

| Multivariate analysis | | | |
| --- | --- | --- | --- |
| Volumetric growth | **Risk factor** | **Odds ratio (95% CI)** | **P value*** |
|  | Symptomatic presentation | 2.401 (0.629-9.160) | 0.200 |
|  | Age at discovery | 0.958 (0.908-1.011) | 0.115 |
|  | Volume at discovery | 1.216 (1.010-1.466) | **0.039*** |
| Surgery | **Risk factor** | **Odds ratio (95% CI)** | **P value*** |
|  | Age at discovery | 1.052 (0.950-1.165) | 0.332 |
|  | Latency period | 1.047 (0.865-1.268) | 0.636 |
|  | Symptomatic presentation | 0.992 (0.033-29.674) | 0.996 |
|  | Volume at discovery | 2.149 (0.701-6.585) | 0.181 |
|  | T2 hyperintensity | 0.030 (0.001-9.814) | 0.235 |
|  | Volumetric growth | 6.632 (0.438-100.515) | 0.173 |

Supplementary table S7: Stepwise multivariate logistic regression analysis of factors associated with meningioma growth, and requirement for surgery. *Significant at P<0.05.
